# Neurocytotoxic effects of iron-ions on the developing brain measured in vivo using medaka (*Oryzias latipes*), a vertebrate model

**DOI:** 10.3109/09553002.2011.584944

**Published:** 2011-07-19

**Authors:** Takako Yasuda, Shoji Oda, Hiroshi Yasuda, Yusuke Hibi, Kazunori Anzai, Hiroshi Mitani

**Affiliations:** 1 Department of Integrated Biosciences, Graduate School of Frontier Sciences, the University of Tokyo, Kashiwa, Chiba; 2 Space Biomedical Research Office, Japan Aerospace Exploration Agency, Tsukuba, Ibaraki; 3 National Institute of Radiological Sciences, Inage, Chiba; 4 Nihon Pharmaceutical University, Ina-machi, Kitaadachi-gun, Saitama, Japan

**Keywords:** High-LET radiation, apoptosis, embryonic brain, medaka, relative biological effectiveness (RBE)

## Abstract

*Purpose:* Exposure to heavy-ion radiation is considered a critical health risk on long-term space missions. The developing central nervous system (CNS) is a highly radiosensitive tissue; however, the biological effects of heavy-ion radiation, which are greater than those of low-linear energy transfer (LET) radiation, are not well studied, especially in vivo in intact organisms. Here, we examined the effects of iron-ions on the developing CNS using vertebrate organism, fish embryos of medaka (*Oryzias latipes*).

*Materials and methods:* Medaka embryos at developmental stage 28 were irradiated with iron-ions at various doses of 0-1.5 Gy. At 24 h after irradiation, radiation-induced apoptosis was examined using an acridine orange (AO) assay and histo-logically. To estimate the relative biological effectiveness (RBE), we quantified only characteristic AO-stained rosette-shaped apoptosis in the developing optic tectum (OT). At the time of hatching, morphological abnormalities in the irradiated brain were examined histologically.

*Results:* The dose-response curve utilizing an apoptotic index for the iron-ion irradiated embryos was much steeper than that for X-ray irradiated embryos, with RBE values of 3.7-4.2. Histological examinations of irradiated medaka brain at 24 h after irradiation showed AO-positive rosette-shaped clusters as aggregates of condensed nuclei, exhibiting a circular hole, mainly in the marginal area of the OT and in the retina. However, all of the irradiated embryos hatched normally without apparent histological abnormalities in their brains.

*Conclusion:* Our present study indicates that the medaka embryo is a useful model for evaluating neurocytotoxic effects on the developing CNS induced by exposure to heavy iron-ions relevant to the aerospace radiation environment.

## Introduction

Astronauts are exposed to a complex radiation field consisting of protons, heavy ions, and secondary particles, including neutrons with a broad range of linear energy transfer (LET) (Badhwar and Cucinotta 1998, Setlow 2003). The dose rate in space is much higher than that of natural radiation on the ground, and the accumulated dose during a long space mission may reach an unacceptable level of health risk. The risks associated with the biological effects of high LET particles in space are still uncertain and need to be quantified to ensure the safety of future interplanetary missions (Yasuda 2009). One of the critical risks for long-term space missions is potential damage to the central nervous system (CNS). Loss of critical cellular components in CNS may lead to performance decrements that could ultimately compromise mission goals and long-term quality of life of astronauts.

Various epidemiological and experimental studies show that the effects of radiation exposure to the CNS are more harmful in the developing embryo and fetus than in the adult organism (International Commission on Radiological Protection [ICRP] 1986, 1992). For humans, epidemiological studies of prenatally exposed survivors of the atomic bombings of Hiroshima and Nagasaki have shown that there may be greater sensitivity for the induction of mental retardation or a reduction in intelligence quotient when exposure occurs during the period 8-15 weeks after ovulation (Otake and Schull 1993, 1998, Schull and Otake 1999). The 8-15-week postovulation period in humans coincides with the period of corticogenesis, when neural cells proliferate rapidly, and these immature neurons migrate from the proliferative zones to their final functional sites in the cerebral cortex (Williams 1989). Many experimental studies in rodents have shown that the most sensitive period for radiation-induced acute cell death is the beginning of the corticogenesis period, namely, embryonic day 13 in the mouse corresponding to embryonic day 15 in the rat (ICRP 2003).

Here, we investigated the effects of high-LET iron-ions on the developing midbrain (optic tectum; OT) using medaka embryos at developmental stage 28 during a period of extreme growth of the OT (Nguyen et al. 1999), as an intact vertebrate model that corresponds approximately to the corticogenesis period in mammals (Ishikawa et al. 2007). The medaka is an ideal model for studying the effects of radiation on the CNS of vertebrates, because the transparency of their eggs and embryos makes it possible to detect morphological abnormalities in the CNS easily using a conventional stereomicroscope (Ishikawa 2000, Shima and Mitani 2004). Moreover, the smaller size of their embryos compared with that of mammalian embryos provides the advantage of easy examination of whole-mount specimens (Furutani-Seiki and Wittbrodt 2004). In addition, we could evaluate the molecular mechanism with regard to neurocytotoxic effects by utilizing the knockout mutants which have been generated by targeting induced local lesions using genome methods (Ishikawa et al. 2010a). Recent genome studies reveal that medaka and mammals share more than 70% of genes (Kasahara et al. 2007, Sasaki et al. 2007). Furthermore, gene expression and histological patterns during brain morphogenesis in medaka are essentially similar to those reported in mammals (Ishikawa et al. 2007, 2008, 2010b), suggesting that many mechanisms underlying brain development are common in all vertebrates (Nieuwenhuys 1998).

Our previous studies showed, by staining with acridine orange (AO), that the developing brain of medaka embryos at stage 28 exhibited transient radiation-induced apoptosis in the marginal proliferating regions in the OT (Yasuda et al. 2008, 2009), and we were able to quantitatively evaluate neurocytotoxic effects of radiation on the developing OT using an apoptotic index. Moreover, medaka have been used to estimate the relative biological effectiveness (RBE) of high-LET radiation based on germ cell mutations (Shimada et al. 2005) and high-energy neutrons measured using apoptotic endpoints in the developing brain and muscle tissue (Kuhne et al. 2009). Based on these findings, we examined the apoptosis in the developing OT of medaka embryos after exposure to high-LET iron-ions using an AO-staining assay. Results obtained in the present study were compared with our previous results from X-ray irradiated embryos (Yasuda et al. 2008, 2009), and the RBE of iron-ions with an LET of 200 keV/μm for the induction of apoptosis in the developing OT was estimated as 3.7-4.2.

## Materials and methods

### Fish and embryos

The fish of an Hd-rR inbred strain, which is established from a southern population (Hyodo-Taguchi and Egami 1985, Hyodo-Taguchi 1996), that have been maintained at the National Institute of Radiological Sciences (NIRS) were used. They were kept at room temperature (26-29°C) under a 14 h light and 10 h dark cycle and fed on a powdered diet (Tetra-min, Tetra Werke, Melle, Germany) once a day to spawn eggs every day.

Egg clusters were rubbed between two small pieces of paper towel to remove filaments on chorions to isolate the eggs. Subsequently, the eggs were incubated in a petri dish containing 7 ml of distilled water containing 10 ^5^% (w/v) methylene blue at 26-29°C to develop. The developmental stages of the embryos were identical to those reported by Iwamatsu (2004).

### Iron-ion irradiation

Iron-ion beams were generated and accelerated using a synchrotron, the Heavy Ion Medical Accelerator in Chiba (HIMAC) at NIRS, in Chiba, Japan. Embryos at stage 28 (30 somite stage, 64 h after fertilization) were irradiated at the entrance of a horizontal iron-ion beams (about 10 cm before the Bragg peak) at room temperature in a specially designed plastic flask filled with distilled water for fitting to a sample holder in the irradiation device (interior dimensions: 35 mm wide, 55 mm high, 2 mm thick). The iron particle energy beam was 500 MeV/nucleon, corresponding to an average LET of 200 keV/μm. Embryos were irradiated with iron-ion beams at doses of 0.2, 0.5, 1.0, and 1.5 Gy, which induced no malformation in the irradiated embryos up to hatching (see Results). Medaka embryos at stage 28 (30 somite stage, 64 h after fertilization) correspond approximately to the early fetal stage embryo of humans (approximately 8-15 weeks postovulation) (Ishikawa et al. 2007).

### Quantification of apoptosis by AO assay

AO (acridinium chloride hemi-[zinc chloride], Sigma-Aldrich, MO, USA), a DNA intercalating vital dye, selectively stains the nuclei of apoptotic cells and does not significantly label necrotic cell nuclei (Abrams et al. 1993, Furutani-Seiki et al. 1996). To quantify the radiation-induced apoptosis in the developing OT at 24 h after irradiation, irradiated embryos were stained with AO and AO-stained rosette-shaped clusters/OT were counted, as described for the evaluation of apoptosis in the X-ray irradiated OT in the methods section of our previous paper (Yasuda et al. 2008).

The AO-stained embryos were observed with a fluorescence microscope (MZFLIII, Leica, Wetzlar, Germany) equipped with an appropriate filter, and their fluorescent images were taken using Fujichrome Sensia 100 daylight film

Differences in the numbers of rosette-shaped clusters/OT at the different iron-ion doses were analyzed using an analysis of variance with Bonferroni post hoc testing. A *P* value of less than 0.05 was considered to be statistically significant and a value of less than 0.01 was considered to be highly significant.

### Histological examination

Medaka embryos at 24 h after irradiation and at the time of hatching (stage 39, 6-7 days after irradiation) were anesthetized and fixed in 4% (w/v) paraformaldehyde in 0.1 M phosphate buffer overnight at 0-4°C. The fixed embryos were dehydrated with ethanol, embedded in plastic resin (Technovit 8100, Heraeus Kulzer, Wehrheim, Germany), and sectioned frontally into a complete series of serial sections (8 μm), as described in our previous paper (Yasuda et al. 2009). The sections were Nissl stained with cresyl violet.

## Results

### Quantification of apoptosis in the irradiated embryos and RBE calculation

**Figure 1:**
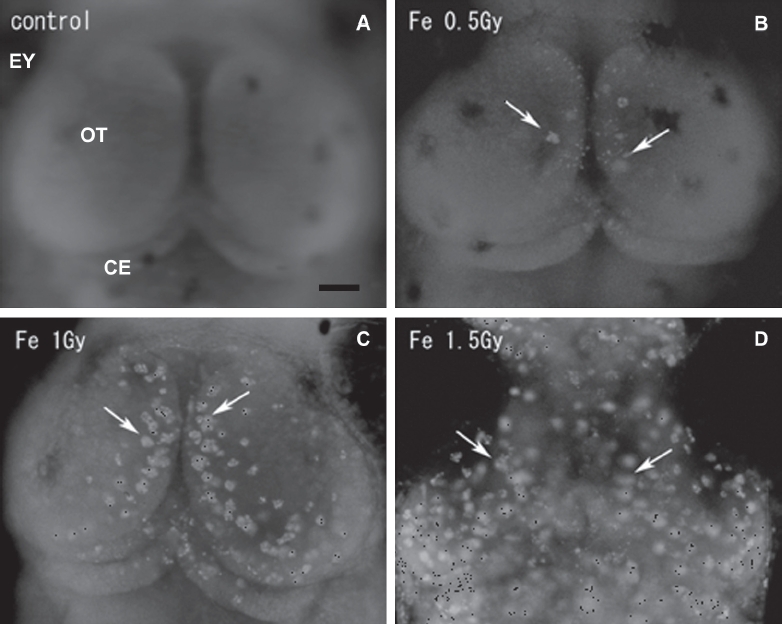
Fluorescence microscopy images of whole-mount AO-stained embryonic brains. The optic tectum (OT) of a nonirradiated normal embryo at stage 30 (A), and those of iron-ion irradiated embryos at 24 h after exposure at a dose of 0.5 Gy (B), 1 Gy (C), and 1.5 Gy (D). Dorsal views, rostral to top. Arrows indicate AO-stained rosette-shaped clusters of apoptosis. OT = optic tectum; EY= eye. Scale bar = 50 μm.

**Figure 2:**
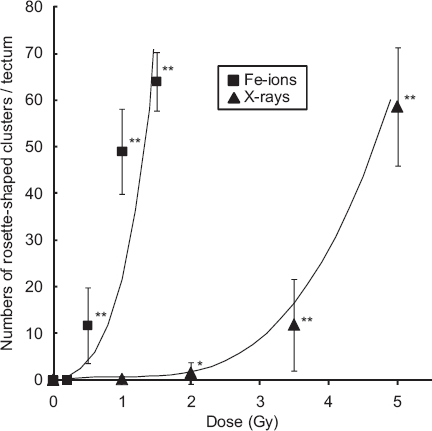
Dose-response curves for the number of rosette-shaped clusters of apoptosis in the optic tectum induced by iron-ions or X-rays. The error bars show the standard deviations of the means. Significant difference is indicated with one or two asterisks at ^*^ *P* < 0.05 and ^**^ *P* < 0.01.

To compare the neurocytotoxicity of low- and high-LET radiation, medaka embryos at stage 28 were irradiated with iron-ions at doses of 0.2, 0.5, 1.0, and 1.5 Gy, and then examined for apoptosis using AO-staining assays at 20-24 h after irradiation, as described for OT apoptosis after X-ray irradiation in our previous work (Yasuda et al. 2008, 2009). Using this method, our previous studies demonstrated two morphologically distinct AO-stained structures (Yasuda et al. 2006, 2008, 2009), namely, AO-positive small single nuclei and rosette-shaped clusters, especially in the marginal area where tectal proliferative zones are present (Nguyen et al. 1999). Our present study showed similar findings of AO-positive rosette-shaped clusters, found mainly in the marginal part of the irradiated OT (arrows in Figure 1), and the numbers of AO-stained rosette-shaped clusters/ OT increased with increasing dose of iron-ions (Figure 2). Exposure to high-LET iron-ion radiation resulted in a dramatically higher induction of apoptosis than that after X-ray irradiation (Figure 2). For example, exposure to only 0.5 Gy of iron-ions induced a similar level of apoptosis (11.7 ± 8.1, *n* = 7) to that resulting from exposure to 3.5 Gy of X-rays (12 ± 9.8, *n* = 12). The numbers of rosette-shaped clusters/ OT in embryos exposed to iron-ions at 0.5 Gy (11.7 ±8.1, *n* = 7), 1.0 Gy (49 ± 9.2, *n* = 7), and 1.5 Gy (64 ± 6.3, *n* = 6) were highly significant compared with control embryos 0 Gy (0 ± 0, *n* = 12). On the other hand, the number of rosette-shaped clusters/OT in iron-ion irradiated embryos at 0.2 Gy (0 ± 0, *n* = 11) was not significantly different from that in control embryos. It is suggested that the threshold dose for the developmental neurocytotoxic effects of high-energy iron-ions is 0.2-0.5 Gy. Thus, irradiation with only 0.5 Gy of high-LET iron-ions produced a highly significant increase in apoptosis compared with control embryos, whereas our previous study of X-ray exposure showed that a dose of 2.0 Gy X-ray radiation was required to achieve this level of significance.

The ratio of iron-ion dose to X-ray dose that produces the same radiation-induced effects in the developing OT, i.e., RBE, was evaluated. No linear, linear-quadratic, or quadratic dose-response curves could be fitted to data from analyses of at least six embryos per dose of iron-ions or X-rays. We therefore searched for a best-fit function that was applicable to both iron-ion and X-ray irradiated embryos. The empirically derived regression curve for iron-ions was determined as y = (x + 0.95)^5^ − 0.774 and that for X-rays was determined as y = (x − 0.9)^3^ + 0.729 (Figure 2). The RBE value of iron-ions relative to X-rays at 15 rosette-shaped apoptotic clusters (y = 15) was estimated to be 4.2 and that at 45 rosette-shaped apoptotic clusters (y = 45) was estimated to be 3.7.

### Histological observation of the developing CNS after exposure to iron-ions

**Figure 3:**
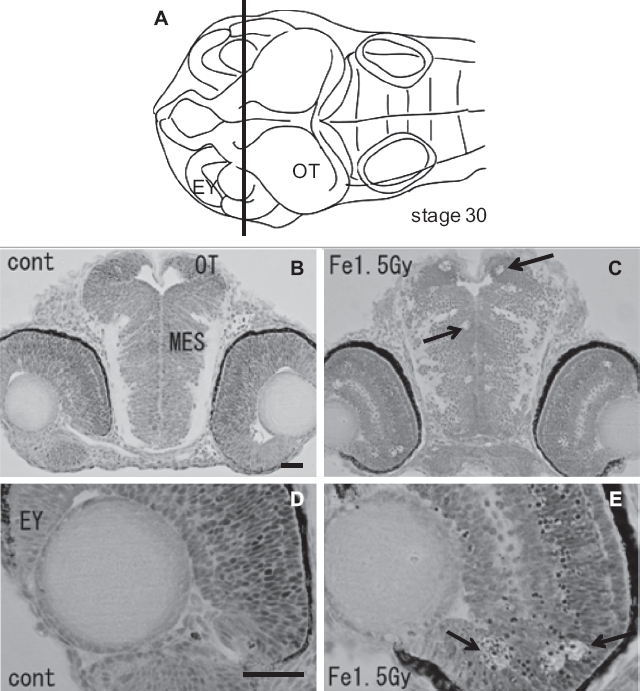
Histology of the iron-ion irradiated embryos at 24 h after irradiation (C, E) and that of nonirradiated embryos (B, D). Dorsal to top. The level is indicated in panel A, which shows the dorsal view of the embryo at stage 30. Arrows indicate clusters of dead cells exhibiting circular holes in the periventricular area of mesencephalon (arrow in C) and in the retinal neuron of the eyes (arrows in E). MES = mesencephalon; OT = optic tectum. Scale bar = 20 μm.

**Figure 4:**
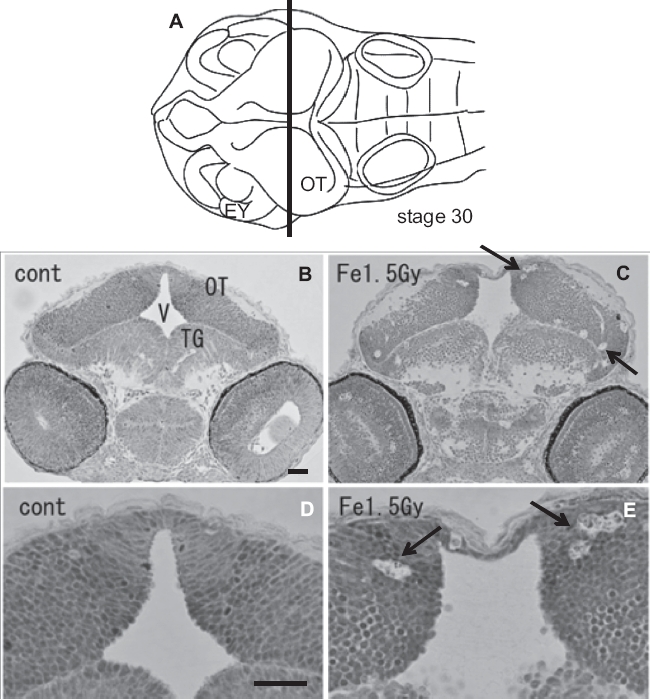
Histology of the iron-ion irradiated embryos at 24 h after irradiation (C, E) and that of nonirradiated embryos (B, D). Dorsal to top. The level is indicated in panel A, which shows the dorsal view of the embryo at stage 30. Arrows indicate clusters of dead cells that appeared as circular holes in the marginal area of optic tectum (arrows in C, E). V = ventricle; OT = optic tectum. Scale bar = 20 μm.

To examine the histological features of AO-stained rosette-shaped clusters, we prepared histological sections of the iron-ion irradiated embryos at 24 h after exposure. The frontal plastic sections of mesencephalon (Figure 3C), eyes (Figure 3E), and OT (Figure 4C, E) showed many clusters of dead cells that appeared as circular holes in the periven-tricular area of the mesencephalon (arrows in Figure 3C), in the retinal neurons of the eyes (arrows in Figure 3E), and in the marginal regions of the OT (arrows in Figure 3C, 4C, E), where the AO-stained rosette-shaped clusters were visualized (arrows in Figure 1). This suggests that these circular holes on the sections are the result of aggregated apoptotic cells, which were demonstrated by electron microscope observations in our previous study (Yasuda et al. 2008).

In our preliminary experiments, most of the irradiated embryos after exposure to iron-ions at a dose of 2.0 Gy hatched normally; however, at a dose of 5.0 Gy, all the irradiated embryos showed embryonic death and severe malformations up to hatching (data not shown). In the present study, after exposure to iron-ions with a dose of 1.5 Gy, almost all the irradiated embryos survived and apparently grew normally (re = 32 out of 35).

**Figure 5:**
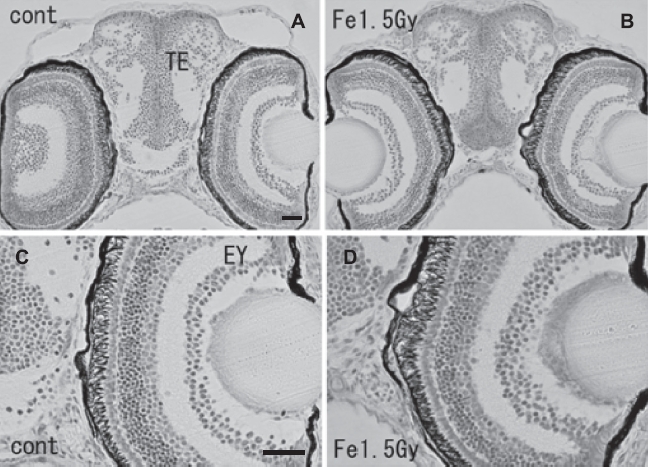
Histology of iron-ion irradiated embryos at the time of hatching (6–7 days after irradiation) (B, D) and that of nonirradiated embryos (A, C). Dorsal to top. Frontal plastic sections at the level of the mid telencephalon (Nissl staining). No abnormal development was detected in the irradiated telencephalon (B) or eyes (B, D). EY = eye; TE = telencephalon. Scale bar = 20 μm.

**Figure 6:**
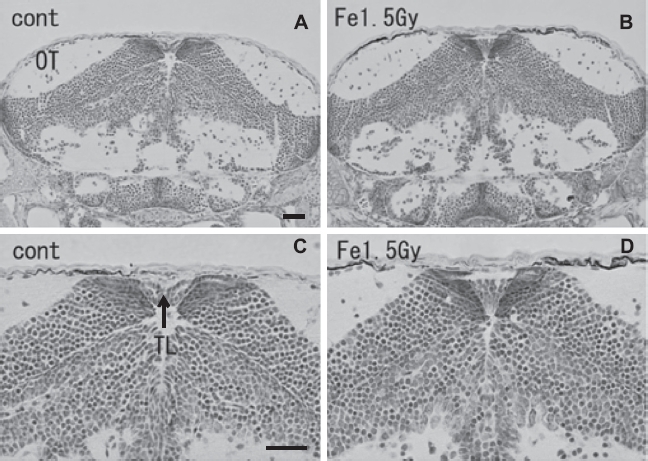
Histology of iron-ion irradiated embryos at the time of hatching (6–7 days after irradiation) (B, D) and that of nonirradiated embryos (A, C). Dorsal to top. Frontal plastic sections at the level of the mid mesencephalon (Nissl staining). No abnormal development was detected in the irradiated optic tectum (B, D). OT = optic tectum. Scale bar = 20 μm.

At the hatching period (8-9 days after fertilization, stages 38-39), frontal plastic sections of the eyes irradiated with 1.5 Gy iron-ions showed no abnormalities such as disorganized laminar arrangement or disordered cell layers in the retinal neurons (Figure 5B, D), where numerous circular holes had been found on sections of irradiated eyes at 24 h after irradiation (arrows in Figure 3C, E). Moreover, histological examination showed that the OT (Figure 6B), the telencephalon (Figure 5B), and the torus longitudinalis (brain subdivision in the midbrain) (Figure 6D) fully developed with no abnormalities.

## Discussion

Astronauts exploring space beyond a low Earth orbit are exposed to cosmic ray flux including high-LET radiation, which has a greater biological effect on tissues than low-LET radiation. The United States National Research Council's Space Studies Board has concluded that further experimentation is essential to determine if CNS damage is a significant risk in terms of estimating health risks for crew members (Setlow 1999, 2003). We have examined the acute biological effects, especially those concerning apoptosis, after exposure of the developing OT to high-LET iron-ions, using intact vertebrate medaka embryos.

In our previous studies, we developed a simple and quick AO-staining assay to detect and quantify apoptotic cells in the irradiated OT (Yasuda et al. 2008, 2009). The response of radiation-induced apoptosis that was observed after exposure to iron-ions using the AO-staining method was similar to that observed after exposure to X-rays in our previous study with respect to its characteristic morphology and position (Yasuda et al. 2008, 2009). Thus, AO-stained rosette-shaped clusters were observed particularly in the marginal OT area (see Results). An in vivo study in rodents showed that the extent and area of acute cell death in the ventricular area of fetal rat brain observed after exposure to 1.5 Gy carbon-ions were similar to those after exposure to 2.0-2.5 Gy X-ray irradiation (Inoue et al. 2000). Furthermore, an in vitro study of neuronal cells showed that changes in morphological characteristics such as apoptosis and cell viability after exposure to carbon-ions were not significantly different from those after exposure to X-rays (Al-Jahdari et al. 2009). These in vivo and in vitro findings showed that the kinds of neurocytotoxic effects on developing neurons resulting from exposure to high-LET radiation do not differ from those resulting from exposure to low-LET X-ray irradiation. Neuronal apoptosis in the irradiated developing OT increased in a dose-dependent manner after exposure to both iron-ions and X-ray radiation, as seen in our previous study (Figure 2). However, the quantitative difference between them was significant. The threshold dose for developmental neurocytotoxic effects after exposure to iron-ions was determined as being 0.2-0.5 Gy, which is a lower dose than that for X-ray-induced damage, as was shown in our previous study of irradiated embryos determined at 1-2 Gy (Yasuda et al. 2008). This finding indicates that prenatal exposure to the iron-ion radiation induced higher developmental neurocyototoxic effects on the developing CNS than did X-ray irradiation.

Almost all the available data indicate that RBE values in vitro and in vivo for cell killing, mutations, and cancer induction in animals increase with LET to a value in the neighborhood of 2-4 at LET values around 100-200 keV/μm (National Council on Radiation Protection and Measurements [NCRP] 1989, Setlow 1999). However, high-LET radiation has a large uncertainty depending on the applied charged-particle species, the type of tissue used and its maturation, and the end-point used. One investigation of the effects of high-atomic number nuclei on tumor induction in the Harderian gland in mice indicated an RBE of 20-40 at high values of LET (Alpen et al. 1993).

The RBE value of iron-ions with an LET of 200 keV/μm in the present study for the induction of apoptosis in the developing OT was estimated to be 3.7-4.2, which was within the range of values reported previously (NCRP 1989, Setlow 1999). An in vitro study of human neuronal progenitor cells (Netra 2) indicated that the RBE value of iron-ions with an LET of 148 keV/μm for the induction of apoptosis was 3.4 (Guida et al. 2005). Both in vivo and in vitro findings showed that exposure to iron-ion radiation induces higher neurocytotoxic effects on the developing brain than low-LET X-rays.

Even though a large number of apoptotic cells were induced in the iron-ion irradiated OT at 24 h after irradiation, histological examination during the hatching period (stage 39, 6-7 days after irradiation) showed no abnormalities in the CNS (Figures 5 & 6). Our present study demonstrated that the iron-ion irradiated embryos could overcome the radiation-induced damage completely during their development, which is in agreement with our previous findings in X-ray irradiated embryos (Yasuda et al. 2009). An in vivo experiment with adult rodents showed that neural precursor cells, immature neurons, in the hippocampal dentate gyrus undergo apoptosis shortly after iron-ion irradiation with 1-3 Gy in a dose-dependent manner (Rola et al. 2004, 2005). Furthermore, no recovery from neuronal damage was observed 3 months after exposure and damage worsened with time up to 9 months later (Rola et al. 2008). This report from rodent studies indicated that high-LET radiation has a significant and long-lasting effect on the neurogenesis. This is obviously in contrast to our present results, which demonstrate a recovery from neuronal damage during development up to hatching. This contradiction is believed to arise for the following two reasons. First, as the radiosensitivity of medaka is much lower than that of mammals (Abrahamson et al. 1973, Ishikawa and Hyodo-Taguchi 1997), the detrimental effects of 1.5 Gy iron-ions on medaka embryos would be smaller than the effects of 1-3 Gy iron-ions on mice. Second, because our present study demonstrated that radiation effects on the CNS of embryo but not on adult fish, the capacity for eliminating damaged neuronal cells and regenerating them with neuronal progenitor cells might be superior (Kriegstein and Alvarez-Buylla 2009) than that seen in 2.5-month-old adult mice (Rola et al. 2008). For eliminating the neuronal damaged cells, it is essential that the dying neurons are quickly phagocytosed by microglia which are resident immune cells of the CNS (Kettenmann 2007, Peri and Nusslein-Volhard 2008). It has been reported in zebrafish embryo that microglia in the embryonic brain at steady and healthy state showed a surprisingly swift wandering behavior (Herbomel et al. 2001). This unexpected behavior of restlessly wandering suggest that microglia in embryonic brain may be constantly patrolling for immune and possibly also developmental and trophic surveillance. This unique behavior of microglia in embryonic brain would result in superior capacity of eliminating damaged neuronal cells in contrast to the adult organism, however, we need further studies about behavior of microglia in irradiated embryonic brain to warrant the capacity of eliminating radiation-induced neuronal damages.

Although histological examinations at the hatching period (stage 39, 6-7 days after irradiation) showed no abnormalities in the CNS in our present study, it is possible that subtle structural changes in the CNS that cannot be detected by histological examination were manifested as behavioral alterations later in life. Behavioral tests are non-invasive measures for the study of alterations after prenatal radiation exposure, and are a sensitive indicator of teratogenic activity (Pecaut et al. 2004, Wang et al. 2007). Experimental data showed that the central dopaminergic system and behaviors mediated by this system are disrupted in iron-ion irradiated rats, as such irradiation induced cognitive declines in spatial learning and memory (Rabin et al. 2000, Shukitt-Hale et al. 2000). These adverse behavioral and neuronal effects are similar to those seen in aged animals, which might be related to an increase in the release of reactive oxygen species (Shukitt-Hale et al. 2000). Moreover, these cognitive declines are associated with specific areas of brain signaling deficits, such as synaptic vesicle proteins, which are important in cognition (Denisova et al. 2002). If these decrements in behaviors also occur in humans, they may impair the ability of astronauts to perform critical tasks. Further investigation to elucidate the effects of embryonic iron-ion irradiation on behaviors found later in adult medaka is warranted in future studies.

To our knowledge, this present study is the first report regarding the effects of high-energy iron-ions on the embryonic brain in vivo using medaka, or any other intact vertebrate, that are relevant to the aerospace radiation environment. Our present results clearly indicate that the AO-staining method is a useful tool for quantifying apoptosis in the developing CNS after exposure to high- and low-LET radiation. Thus, medaka embryos are a useful model for investigating embryonic neuronal damage associated with high- and low-LET radiation.
